# Differences in inhibitory control and motor fitness in children practicing open and closed skill sports

**DOI:** 10.1038/s41598-021-82698-z

**Published:** 2021-02-17

**Authors:** Damiano Formenti, Athos Trecroci, Marco Duca, Luca Cavaggioni, Fabio D’Angelo, Alberto Passi, Stefano Longo, Giampietro Alberti

**Affiliations:** 1 Department of Biotechnology and Life Sciences (DBSV), University of Insubria, via Dunant 3, 21100 Varese, Italy; 2 Department of Biomedical Sciences for Health, Università Degli Studi Di Milano, Via Kramer 4/A, 20129 Milan, Italy; 3 Obesity Unit and Laboratory of Nutrition and Obesity Research, Department of Endocrine and Metabolic Diseases, IRCCS Istituto Auxologico Italiano, Milan, Italy; 4 Department of Medicine and Surgery (DMC), University of Insubria, via Dunant 5, 21100 Varese, Italy

**Keywords:** Cognitive neuroscience, Health policy, Paediatrics, Quality of life

## Abstract

The aim of the present study was to investigate the differences between types of sport (i.e., closed vs. open skills sport) on inhibitory control and motor fitness in children. Forty-nine children were allocated into three groups based on their sports participation, which comprised an open skill sport group, a closed skill sport group, and a sedentary group. Participants were tested on cognitive performance (inhibitory control by the Flanker task) and motor fitness (reaction time, speed, agility, power, balance). Open skill sport group appeared to display higher inhibitory control (response time and accuracy of incongruent condition of the Flanker task) and motor fitness performance (reaction time, speed, agility, power) than sedentary group, whereas its superiority over closed skill sport group was found only in speed and agility. Moreover, closed skill sport group had only a better reaction time than sedentary group. Our data supports the framework according to which cognitive demands in complex motor actions may contribute to explain the beneficial effects of exercise on inhibitory control. This might suggest that the complexity of the environment (typical in open skill sports) in which sport training is performed plays a key role for both cognitive and motor development in children.

## Introduction

The benefits of physical activity across the lifespan are not restricted to physical health but are also extended to cognitive functions, which refer to the mental abilities involved in the processes underlying perception and action. Indeed, a recent systematic review and consensus statement has provided evidence of positive associations among physical activity, fitness, cognition, and academic achievement in children^1^. Additionally, cross-sectional studies have suggested that children and adolescents with higher physical fitness or those participating in regular physical activity have superior cognitive functions compared to those with lower physical fitness, or those who are sedentary^2–6^.

In this context, it has been suggested that the improvements in cognitive functions through physical exercise may be somehow related to the characteristics of exercise movements^7,8^. The interaction of these characteristics with the environment has led to classify sports into open and closed skill sports^9–11^. In open skill sports, participants are required to perform in a dynamically changing, unpredictable, and externally-paced environment, adapting their responses to external stimuli (e.g., team and ball sports, combat sports). In contrast, closed skill sports are characterized by a relatively constant, predictable and self-paced environment, in which motor movements follow repeated and predetermined patterns (e.g., running, swimming, cycling).

During childhood, children may decide to participate in either open or closed skill sports. Consequently, due to the different demands of open and closed skill sports, this choice might contribute to influence both their physical fitness and cognitive development. Physical fitness is a multidimensional concept including both health-related (cardiovascular fitness, body composition, strength and muscular endurance, flexibility) and skill-related components. The latter, also denoted as motor fitness^12^, involves the ability to learn and perform motor skills (reaction time, speed, agility, power and balance), which may be associated also with cognitive performance in the domains of perceptual speed and executive functions^13^.

It has been argued that cognitively demanding physical activity may be beneficial for enhancing cognitive functions in children^14,15^. Coordinatively demanding and nonautomated activities (typical of open skill sports) activate the same brain regions that are used to control cognition^16,17^. A typical situation developing in open skill sports requires to suppress inappropriate actions (denoted as inhibitory control^18^) that plays a pivotal role for selecting correct behaviours in both sports^19^ and daily-life activities^20^. In accordance, recent studies highlighted the importance of inhibitory control to discriminate the players’ competitive level in open skill sport (volleyball)^21^, and that open skill sports athletes performed better than those with a closed skill sport background (e.g., track-and-field, swimming) in cognitive tasks assessing inhibitory control^20^ and other cognitive performance components^2,20,22^. A systematic review on this topic tends to support that open skill sports are more effective for improving some aspects of cognitive functions (including inhibitory control) compared to closed skill sports^8^. However, whether or not the practice of a type of sport might influence inhibitory control and whether or not this might be associated with motor fitness in children remains to be elucidated.

Therefore, the aim of the present study was to investigate the differences between types of sport (i.e., open vs. closed skills sport) on inhibitory control and motor fitness in children. We hypothesized that individuals practicing open skills sports would perform better than controls not engaged in any sport activity, and those practicing open skills sports would exhibit superior inhibitory control compared to participants practicing closed skills sports.

## Results

Table 1 shows demographic characteristics of the three groups. Non-significant differences between the three groups were found for age, height and body mass. The ANOVA revealed significant differences among groups in physical activity (F_(2,46)_ = 218.55, *p* < 0.0001). Post-hoc analysis showed that physical activity was significantly higher in OSG than SG (*p* < 0.0001, *d* > 2) and in CSG than SG (*p* < 0.0001, *d* > 2).

**Table 1:** Demographic characteristics of OSG (open skill group), CSG (closed skill group) and SG (sedentary group).

Groups	Age (years)	Height (m)	Body mass (kg)	Maturity offset (years)	Physical activity (h/week)
OSG (n = 17)	10.57 ± 0.45	1.40 ± 0.07	35.35 ± 6.19	-2.7 ± 0.9	3.58 ± 0.53
CSG (n = 16)	10.31 ± 0.38	1.39 ± 0.04	35.67 ± 6.90	-2.1 ± 0.8	3.56 ± 0.35
SG (n = 16)	10.22 ± 0.59	1.37 ± 0.09	36.57 ± 9.69	-2.1 ± 1.0	0.75 ± 0.40***

Values are shown as mean ± SD.

***p < 0.0001 vs. CSG and OSG.

**Figure 1:**
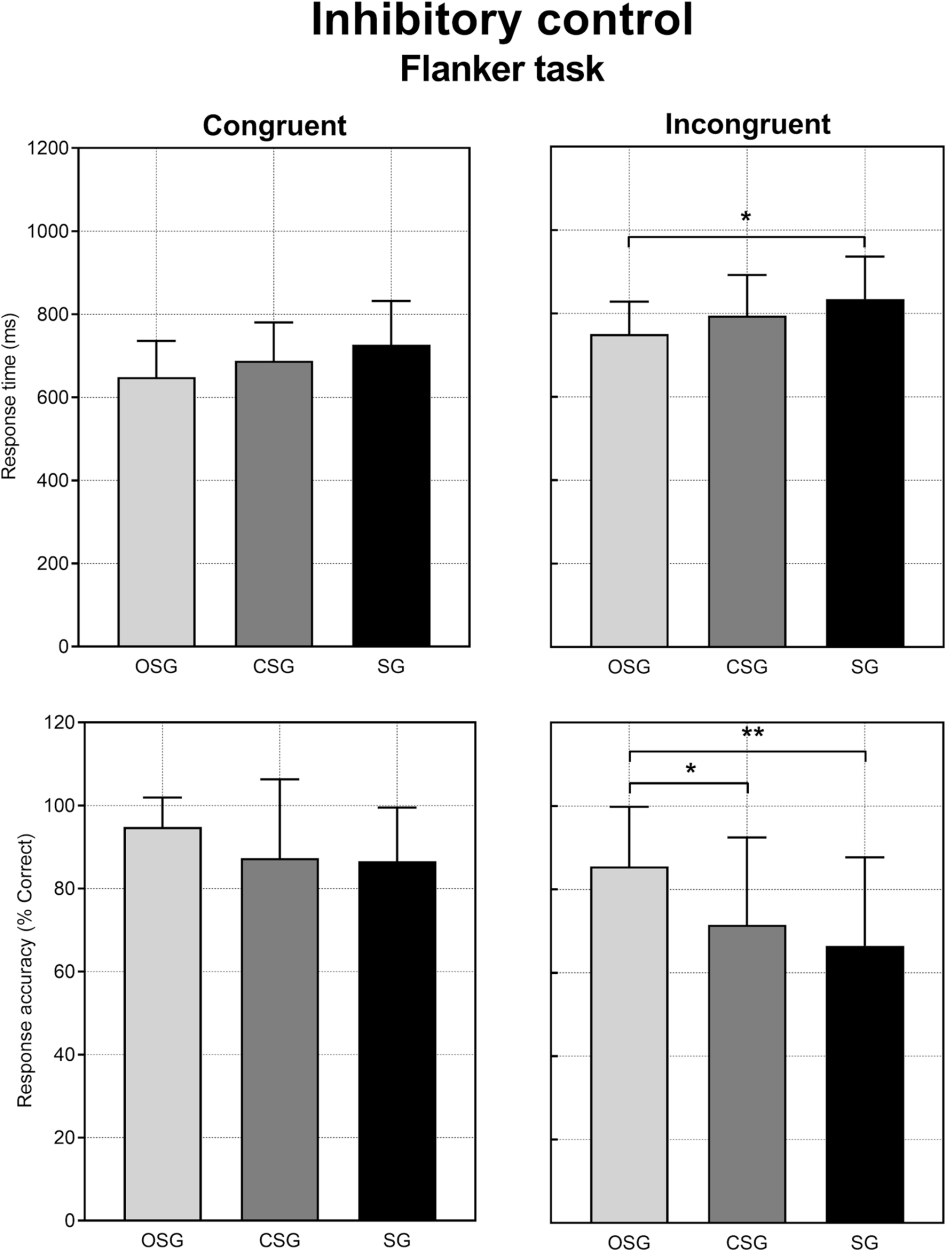
Cognitive performance reflecting inhibitory control of the Flanker task in the three groups. OSG: open-skill group, CSG: closed-skill group, SG: sedentary group. Values are shown as mean ± SD. Significant differences obtained from pairwise comparisons: *p < 0.05; **p < 0.01.

Cognitive performance in the Flanker task is shown in Fig. 1. Although there was not a significant interaction (group × Flanker condition) in response time (F_(2,46)_ = 0.042, *p* = 0.95), a significant main effect of Flanker condition (F_(1,46)_ = 135.2, *p* < 0.0001) and a significant main effect of group (F_(2,46)_ = 3.497, *p* = 0.038) were observed. Post-hoc analysis showed that OSG presented lower response time in the Incongruent condition than SG (*p* = 0.034, *d* = 0.93). Similarly, despite the lack of significant interaction (group × Flanker condition) in response accuracy (F_(2,46)_ = 2.997, *p* = 0.05), a significant main effect of Flanker condition (F_(1,46)_ = 68.50, *p* < 0.0001) and a significant main effect of group (F_(2,46)_ = 3.676, *p* = 0.033) were observed. Post-hoc analysis showed that OSG presented better accuracy in the Incongruent condition than SG (*p* = 0.004, *d* = 1.05) and CSG (*p* = 0.033, *d* = 0.78).

**Figure 2:**
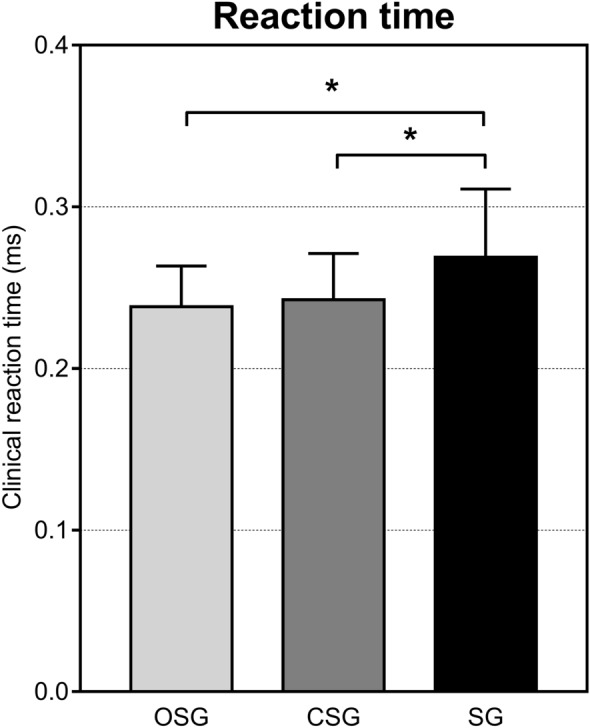
Reaction time assessed by the Clinical reaction time test in the three groups. OSG: open-skill group, CSG: closed-skill group, SG: sedentary group. Values are shown as mean ± SD. *p < 0.05.

Reaction time is shown in Fig. 2. The ANOVA revealed significant differences among groups in the clinical reaction time (F_(2,46)_ = 4.15, *p* = 0.022). Post-hoc analysis showed that OSG and CSG presented lower clinical reaction time than SG (*p* = 0.03, *d* = 0.89; *p* < 0.05, *d* = 0.72, respectively) whereas no differences were observed between OSG and CSG (*p* = 0.707, *d* = 0.02).

**Figure 3:**
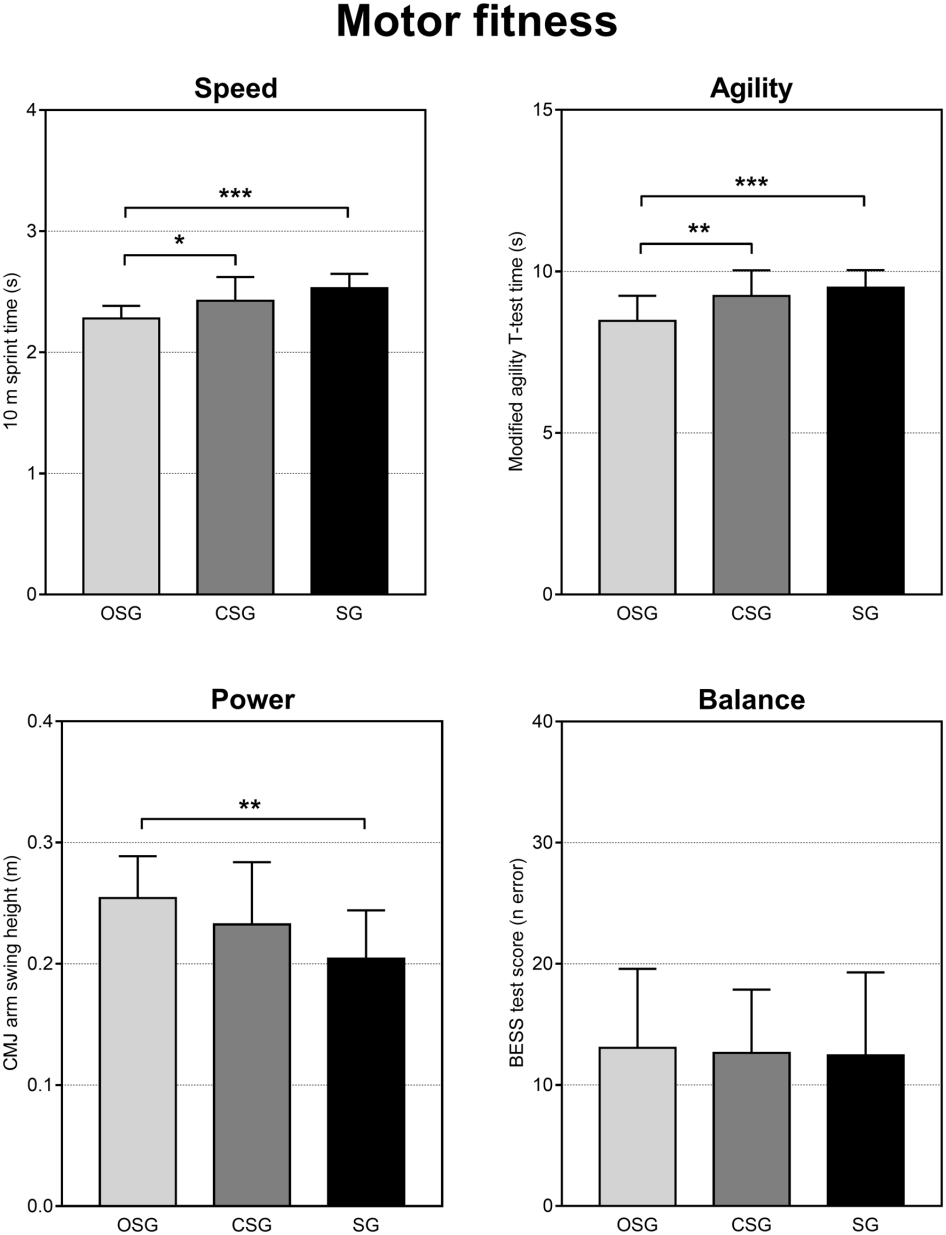
Motor fitness outcomes (speed, agility, power, balance) in the three groups. OSG: open-skill group, CSG: closed-skill group, SG: sedentary group. Values are shown as mean ± SD. *p < 0.05; **p < 0.01; *p < 0.001.

Motor fitness is shown in Fig. 3. The ANOVA showed significant differences among groups in speed (F_(2,46)_ = 12.31, *p* < 0.0001). Post-hoc analysis revealed that OSG was faster in the 10-m sprint than SG (*p* < 0.0001, *d* = 2.26) and CSG (*p* = 0.011, *d* = 0.94), and CSG was faster than SG (*p* = 0.04, *d* = 0.60). One-way ANOVA revealed significant differences among groups in agility (F_(2,46)_ = 9.24, *p* = 0.0004). Post-hoc analysis showed that OSG was faster in the modified agility T-test than SG (*p* < 0.001 *d* = 2.26) and CSG (*p* = 0.006, *d* = 0.94), whereas CSG and SG were not different (*p* = 0.31, *d* = 0.60). Concerning power, the ANOVA showed significant differences among groups (F_(2,46)_ = 5.79, *p* = 0.005). Post-hoc analysis showed that OSG presented better jump performance in the CMJ with arm swing test than SG (*p* = 0.004, *d* = 1.41), whereas no differences were found between OSG and CSG (*p* = 0.145, *d* = 0.48), and between CSG and SG (*p* = 0.125, *d* = 0.66). For balance, one-way ANOVA did not reveal significant differences among groups in the BESS test score (F_(2,46)_ = 0.042, *p* = 0.95).

## Discussion

The main finding of the present study was that OSG seemed to present higher inhibitory control (response time and accuracy of the incongruent condition of the Flanker task) and motor fitness (Clinical reaction time, 10-m sprint, modified agility T-test, CMJ arm swing) than CSG and SG. Regarding the Flanker task, our hypotheses were partially confirmed as OSG showed higher accuracy than both CSG and SG while displaying shorter response time than SG in the incongruent condition. These findings demonstrated that practicing open skill sport activities, rather than closed skill sport activities, may be associated with a higher performance in both cognitive and motor fitness tests than being sedentary. This suggests that the type of environment in which sport is performed plays a key role for both cognitive and physical development in children.

The present study specifically found connections among types of sport activities and both motor and cognitive performance (inhibitory control). As childhood is a sensitive period in the acquisition of motor skill development^23^, encouraging participation in activities within a complex sport environment may be useful for promoting links within brain areas involved in both cognitive and motor skills^8,24^. Concerning cognitive performance, no interactions (group × Flanker condition) were observed for both response time and accuracy of the Flanker task. When looking at the post-hoc analyses of the main effects, OSG demonstrated shorter response time than SG and higher response accuracy than CSG and SG in the incongruent condition. The fact that OSG presented better performance than SG in the incongruent, rather than congruent condition, was almost expected. Given their unpredictable nature, the open skill sports require a high level of inhibitory control compared with closed-skill sports reflecting the incongruent conditions with distractors^25^. With regards to CSG, our data revealed no inhibitory control advantage on closed skill sport participants, who had similar response time and accuracy to sedentary group, despite higher level of physical activity (Table 1). Altogether, these findings appear consistent with those reported in interventional studies showing that open skill exercises resulted in larger improvement in cognitive performance than closed skill exercises in primary school children^14,26^. However, it has to be recognized that the present lack of group × Flanker condition interactions imposes caution when generalising on the higher OSG performance in the incongruent condition compared to the other two groups.

The notion that sports modality may affect cognitive performance was also supported by previous cross-sectional studies in both university students^20^ and primary school children^2^. The latter showed that children regularly participating in an open skill sport activity (football) had better vigilance performance than children participating in a closed skill sport activity (track-and-field) and non-athletic controls, whereas no significant difference was observed between closed skill athletes and sedentary controls^2^. It is worth noticing that in a regression model proposed by those Authors, cardiovascular fitness was not a significant predictor of cognitive performance. This seems to suggest that the superiority of football players compared to track-and-field athletes and sedentary controls might not be mediated by cardiovascular fitness exclusively, nuancing the cardiovascular fitness hypothesis^2^. Rather, it has been suggested that cardiovascular fitness should not be considered as the exclusive responsible of the cognitive benefits of exercise, but it likely plays an important role together with the cognitive component of exercise (cognitive stimulation hypothesis)^2,27^.

In accordance, studies have demonstrated that not only cardiovascular fitness, but also skill-related components of physical fitness were associated with cognitive benefits in children^28–30^. These results were supported by neurophysiological studies showing underlying neural mechanisms that contributed to explain the relationship between motor fitness and cognition^31,32^. For example, in one study^32^ individuals showing a high level of motor fitness demonstrated a better inhibitory control compared to peers of low level of motor fitness. This was accompanied by an increased activation of task-specific areas involving executive and visual-spatial processing on one hand, and by a less compensatory over-activation of frontal areas on the other (fewer resources were needed to perform the task assessing inhibitory control)^32^. Moreover, common regions of the cortex were activated during both motor tasks and cognitive tasks, which include the prefrontal cortex (important for executive functions) and the cerebellum (important for complex movements)^31^. These notions point to the conclusion that an association between complex motor activities and cognitive performance exists^13^. Accordingly, our data indicated that practicing open skill sports might impact favourably not only motor fitness benefits, but also the domain of inhibitory control of executive functions in children.

Concerning motor fitness, it is interesting to note that reaction time was the only variable where CSG obtained similar results as OSG (Fig. 2). This is in agreement with a previous study that showed similar reaction time in individuals practicing open and closed skill sports and may be due to similar visual-cognitive skill of both groups in adult athletes^33^. For the other parameters, OSG showed better agility and speed skills compared to CSG and SG (Fig. 3). Similarly, the superiority of OSG over SG was also found in power (although no significant difference was found between OSG and CSG), whereas no significant differences among groups were observed in balance (Fig. 3).

It is well known that closed skill sport activities are performed in a relatively stable and predictable environment, in which motor actions are repeated regardless of the external context^9^. On the contrary, as performed in a dynamic and changing environment, open skill sport activities are mainly characterized by sport-specific motor actions that must be continually adapted in response to external stimuli^9^. In this perspective, the task complexity environment-related typical of open skill sports may have a pivotal role in the development of both cognitive and motor skills^8,31^. This shifting context of open skill sports, in which inappropriate actions have to be suppressed, may be related to greater challenge of motor skills and activation in brain systems involving executive functions (especially the prefrontal cortex)^16,34^. In this wake, it is plausible that children participating in open skill sports may have the opportunity of engaging and stimulating their motor skills, that, in turn, may contribute to the development of executive functions. Summarizing, our findings support the conceptual framework according to which cognitive demands in complex motor actions, typical of open skill sports, may contribute to explain the beneficial effects of exercise on cognitive performance^8,16,35^.

The current study presents limitations that should be acknowledged. First, as a cross-sectional study aiming at investigating potential differences in inhibitory control and motor fitness between children participating in different sport activities, we cannot fully exclude the possibility that children with better executive functions and motor fitness would have chosen to participate in open skill sports. Therefore, we put in evidence that further investigations are warranted to confirm the findings of the present study, especially randomized controlled trials assessing causal effect of types of sport participation on cognitive and motor skills. Further, our sample was composed by children attending the same school. It is possible that recruiting children from different schools and environments would have been more informative on the processes related to motor and cognitive skills. Additionally, it should be pointed out that measures such as aerobic fitness, intelligence quotient and socioeconomic status are associated with cognitive performance across the lifespan^36–38^, representing potential confounding factors. Hereby, further studies are warranted to contemplate such confounders in the attempt of advancing knowledge in the field of exercise modality-cognition interaction.

In conclusion, although more research is needed to deeply understand the association between sport participation and inhibitory control, our findings may contribute to advance knowledge on the role of youth sports associated with cognitive development. Our results suggest that environment characteristics in which sport training is performed plays a key role for both cognitive and motor development in children. Although caution should be applied, evidence from the current study extends the knowledge from previous literature that open skill sport activities may have superior benefits for developing cognitive and motor skills than closed skill sport activities. Engagement in motor actions in response to complex environmental stimuli (as open skill sports) stimulates brain regions responsible for the development of executive functions and motor fitness^16^. Therefore, it is recommended that public health system administrators implement policies to promote sport participation for children both inside and outside the school context, with special emphasis on activities targeting not only health-related components of physical fitness, but also motor fitness.

## Methods

### Participants

Forty-nine middle-age children (21 males: 10.36 ± 0.49 years; 28 females: 10.32 ± 0.49 years) were recruited to participate in the present study from an elementary local school. The school board and physical education teachers gave the approval. An overview of the participants’ characteristics is shown in Table 1. The participants were allocated into three groups based on their regular sports participation, which comprised an open skill sport group including soccer, basketball, volleyball, and martial arts (OSG, n = 17), a closed skill sport group including rhythmic gymnastics, swimming, and classical ballet (CSG, n = 16), and a sedentary group (SG, n = 16) with no historical specialty in any sport/exercise. OSG and CSG reported more than 3 years of systematic sport participation with at least 3 h of training per week. CSG reported no regular sport participation out of school (1 h or less per week). All participants and their parents were informed about the aim of the study. Parents or legal guardians provided written informed consent before the investigation. The study was approved by the Ethics Committee of the Università degli Studi di Milano, in accordance with the Declaration of Helsinki.

### Procedures

Before experimental testing sessions, participants underwent a familiarization session to get accustomed with all testing procedures. After three days, participants took part in a testing session to assess anthropometric characteristics and cognitive performance (Flanker task). Regarding anthropometric characteristics, height, sitting height and body mass were recorded. The corresponding maturity offset of each participant was computed by the equation of Mirwald and Colleagues^39^ (Table 1). After 48 h, participants underwent a second testing session to assess motor fitness (reaction time, speed, agility, power, balance).

### Cognitive performance

#### Inhibitory control

Inhibitory control was assessed using a computer-based task with a modified version of the Flanker task with arrows^40^. Participants were requested to respond as quickly and accurately as possible to the direction of a left or right target arrow while ignoring two flanking arrows on each side pointing in the same or the opposite direction. The task included two different conditions. The congruent condition consisted of trials in which both the target arrow and the flanking arrows pointed in the same direction (left: <<<<< or right: >>>>>). The incongruent condition consisted of trials in which flanking arrows pointed in the opposite direction of the target arrow (<<><< or >><>>). Participants were requested to press the button A of the keyboard when the target arrow pointed to the left and to press the button L when the target arrow pointed to the right. The 100 trials presented within the task were distributed equally among the two experimental conditions (50 for congruent and 50 for incongruent condition) and were randomized. Participants had 2 s to respond from the visual stimulus presentation. In case of response times shorter than 200 ms, they were excluded from the analysis. Mean response time of the correct responses and response accuracy were computed for both congruent and incongruent condition separately and considered for the analysis.

### Motor fitness

#### Reaction time

Simple reaction time was assessed using the clinical reaction time test^41^. Participants sat on a chair with their dominant forearm and wrist on a table, maintaining their open hand at the edge of the table. The examiner suspended the clinical reaction time apparatus vertically in a way that the weighted disk was aligned with the open hand of the participant. The examiner released the apparatus at random time intervals (from 4 to 10 s) and the participant was requested to catch it as quickly as possible, maintaining his gaze on the weighted disc. Gazing at the examiner’s hand was not allowed. The distance from the top of the disk to the most superior part of the participant’s hand was recorded. This distance was then converted to clinical reaction time in milliseconds using the equation of time needed for an object to fall a given distance. After three practice trials, participants performed eight experimental trials, and their mean value was considered for the analysis.

#### Speed

The test consisted of three maximal sprint bouts separated by 2 min of recovery. All participants started from a standing position with a free departure^42^. The best performance time was used within the analysis. Each sprint bout was recorded by an electronic timing gates system (Witty, Microgate, Bolzano, Italy) fixed at 0.6 m above the ground and placed 0.3 m back from the starting line.

#### Agility

The modified agility T-test was used to assess agility (specifically, the physical component of agility)^43^. Participants began with both feet behind the starting line. At subjective discretion, they sprinted forward for 5 m to the central cone touching it with the right hand. Facing forward and avoiding to cross feet, they shuffled to the left for 2.5 m touching the left cone with the left hand. Then, they shuffled to the right for 5 m touching the right cone with the right hand. After that, they shuffled back to the left touching the central cone. Finally, they ran backward as quickly as possible returning to the starting line. Participants who crossed one foot in front of the other, failed to touch the cones, or failed to remain facing forward was requested to repeat the test after a complete recovery. Participants performed three trials separated by 3 min of recovery. The best performance time was considered in the analysis. Performance time was recorded using a timing gate system (Witty; Microgate, Bolzano, Italy).

#### Power

Power ability of the lower limbs was assessed using countermovement jump test with arms swing (CMJ). The Optojump next system (Microgate, Italy) was used to measure vertical jump height of each participant. Participants performed 3 jumping trials and the best performance was used for the analysis. A recovery of 3 min was given between each trial.

#### Balance

The balance error scoring system (BESS) test was employed to assess the static balance of the participants^44,45^. The participants were instructed to maintain three stances (bilateral, unilateral and tandem stances) on both a firm and foam pad. In the bilateral stance, the feet were flat with the internal *malleoli* closed to each other. In the unilateral stance, the body was supported by the non-dominant limb, and the dominant limb off the ground with the hip and knee flexed at 20° and 45° in the frontal plane, respectively^46^. Angles were checked through a manual goniometer. In the tandem stance, the foot of the dominant limb was positioned in front of the non-dominant one with the latter touching the heel of the dominant foot by the toe. Each participant was instructed to keep a stable position for 20 s in each stance. Then, the number of errors detected during each stance was recorded in line with previous protocols^45,46^. In case of multiple errors at once, they were counted as single error. The BESS test score was obtained by summing the total errors recorded on the firm and foam pad surfaces.

### Statistical analysis

The normality of the distributions was assessed by the Shapiro–Wilk’s normality test. All variables met the assumption of normality. One-way analysis of variance (ANOVA) was used to investigate differences between the three groups for each variable with the exception of inhibitory control. A two-factor (i.e., group and Flanker task condition) mixed-model ANOVA (between-factor: group, three levels; within factor: Flanker task condition, two levels) was used to test whether inhibitory control (response time and response accuracy) was different between the three groups. Specifically, both interaction and main effects were analysed. In case of significance, Holm-Sidak’s multiple comparisons test was used for post-hoc analysis. Cohen’s *d* (*d*) effect size was computed to assess the magnitude of the difference. Each *d* value was classified as *small* (0.20 < *d* < 0.49), *medium* (0.50 < *d* < 0.79), and *large* (d ≥ 0.80) effect. A *p*-*value* lower than 0.05 was considered statistically significant. Values are shown as mean ± SD. Statistical analysis was performed using GraphPad Prism version 7.00 for Windows (GraphPad Software, San Diego, CA, USA).

## Data Availability

The datasets generated during the present study are available from the corresponding author on reasonable request.
